# Investigating dynamics of lentiviral vector secretion from HEK293T producer cells using a fractionated perfusion system

**DOI:** 10.1002/biot.202300097

**Published:** 2023-09-21

**Authors:** Lauren M. Timmins, Patrick Erickson, Biju Parekkadan

**Affiliations:** 1 Department of Biomedical Engineering, Rutgers University, Piscataway, New Jersey, USA; 2 Department of Chemical and Biochemical Engineering, Rutgers University, Piscataway, New Jersey, USA; 3 Department of Medicine, Rutgers Biomedical Health Sciences, New Brunswick, New Jersey, USA

**Keywords:** gene therapy, HEK-viral interactions, stable viral production, vector manufacturing

## Abstract

Mammalian cell culture is quickly becoming the go to engineering vehicle to mass produce viral vectors in a manner that is safe, convenient, reproducible, and cost and scale effective. Human embryonic kidney (HEK293) cells, in particular, have been utilized and customized (via differentiated transgene expression, modified culture parameters, addition of cytostatic culture agents) to increase vector yields. However, less attention has been made to understanding innate processes within the cells (such as, immune response, cell cycle, metabolism) themselves to better control or increase viral vector product yield. Accordingly, herein, the variation in viral production was studied from HEK cells over time using a one-way perfusion system and bioreactor to study the impact of external factors on secretion dynamics without retrotransduction. Specifically, the impact of cell density on viral titer, transduction efficiency, and LDH, was studied. Next, we look at the impact of using an inflammatory reporter cell line on viral output, and the secretion dynamics from HEK cells when we use sodium butyrate (cell cycle arrest agent). Lastly, we assess how downregulation of the PDK pathway increases viral titer. Altogether, we investigated the impact of various interventions to increase transient protein expression and viral output from HEK cells in a controlled and measurable environment to ultimately increase the efficiency of HEK cells for downstream clinical applications.

## 1 | INTRODUCTION

The biopharmaceutical industry has progressed immensely with the development of what can be called “engineering biology.” Specifically, engineering biology is a set of methods used to design, build and test engineered biological systems used to process materials (in this case human materials (cells)). The use of mammalian cell cultures as a production vehicle for viral vaccines and viral vectors in a safe, flexible, fast, and cost-effective manner has dramatically changed how life saving medicines, including cell-based medicines, have been made.^[1,2]^ In particular, human embryonic kidney cells (HEK293) have been used for over 35 years for production of viral vectors for cell and gene therapy and have outperformed other cell types as a platform in many areas. HEK293 cells are easy to grow in serum-free suspension culture which enables large-scale production, have fast doubling times, are amenable to transfection and have high efficiency of protein production.^[3]^ Importantly HEK293 cells are of human origin, meaning that they are a suitable vessel for producing human biotherapeutics.^[4–6]^ Research in the area of large scale biomanufacturing using HEK293 cells has been focused on finding new ways to improve the efficiency of transient protein expression in HEK293 cells. Many variations of the HEK293 cell line have been established, most notably the HEK293T cell line expressing the large T-antigen which considerably improves the protein expression levels during transient transfection.^[4,7]^ Other variations such as HEK293S, HEK293E, HEK293F, and many more have improvements including genetic integration of vector components, adaptability to suspension cultures and growth in defined medium.

External process parameters, such as media composition, expression vector type and size, transfection reagents or other chemical agents and culture conditions have been investigated to improve the recombinant protein yields from HEK293 cells.^[4,8,9]^ There are several transfection reagents that have been used for increased viral production in particular, including polybrene, protamine sulfate, polyethylenimine, and lipofectamine 3000. The choice and modification of the expression vector promotor, including CMV, EF1*α*, and PGK, is also important for production of lentiviral or other viral vectors due to having a direct impact on robust transgene expression and targeted gene delivery in vivo.^[10–12]^ Additionally, culture parameters such as temperature, CO_2_, and O_2_ have been optimized.^[9]^ While much effort and focus has been placed on optimizing the expression vectors, media and culture conditions, and transient transfection protocols, less effort has been attributed to understanding specific cellular features of HEK293 cells that would be required for high productivity of protein production.^[13]^ Specifically, cytostatic culture agents, such as sodium butyrate, trichostatin A, valproic acid, and dimethyl sulfoxide, have all been used to enhance production in HEK293 cells by slowing cell growth rate.^[8,14,15]^ Researchers have demonstrated upward boosts in yield from 200 mg L^−1^ in adherent systems to 140–6600 mg L^−1^ in suspension systems of protein product yields when using these techniques (stable clone expression).^[16,17]^ Other examples of techniques used to enhance transient gene expression and protein production titer in producer cell lines include hyperosmolarity and expression of cell cycle regulators.^[3,15,18–20]^

**FIGURE 1:**
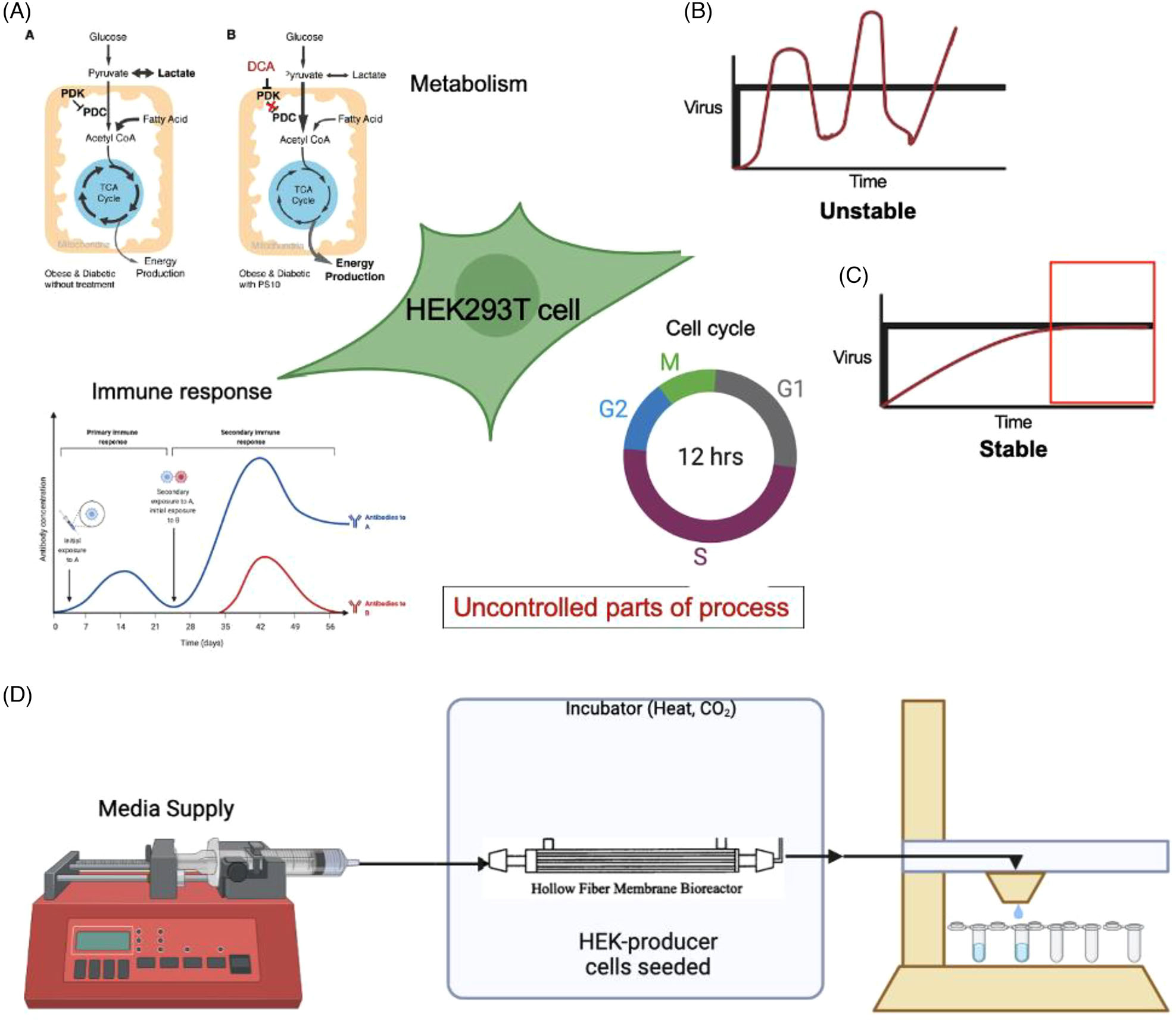
(A) Intracellular immune response, cell cycle, and metabolism are processes in HEK cells that can be modified to impact of production of viral vectors. (B and C) Viral production from HEK cells is not always stable over a long-term cultivation period. (D) Variation in viral production from HEK cells can be studied over time using a one-way perfusion system enabling the study of secretion dynamics without retrotransduction.

Despite the time-dependence inherent to the transient gene expression used to manufacture lentiviral particles, the time course of the secretion of vectors from producer cells is rarely characterized. The hour-scale variations in secretion rates present in these systems are difficult to measure over multiple days due to the need for frequent sample collection. In this report, we utilize a unique one-way perfusion system illustrated in Figure 1D to study secretion dynamics of lentiviral vectors from HEK cells seeded in hollow fiber bioreactors. In this system, cells are seeded in the outer lumen space where they adhere to the semipermeable membranes of the hollow fibers. Secreted viral particles permeate through these membranes into the inner lumen space where constantly flowing media removes them to a fraction collector which automatically distributes the effluent stream into a series of fractions over time, enabling the measurement of changes in secretion rate. Using this system, we investigated the impacts of cell number, inflammatory response, cell cycle arrest, and pyruvate dehydrogenase complex downregulation on the time course of viral vector secretion (Figure 1A–C), providing a novel view into the vector production process and how these different interventions play out over time.

## 2 | MATERIALS AND METHODS

### 2.1 | Cell culture

All cell cultures and bioreactors were kept in humidified incubators at 37°C and 5% CO_2_. Human embryonic kidney cells expressing the SV40 large T antigen (HEK293T) (ATCC: CRL-3216) were grown in DMEM/F12 medium (Gibco) supplemented with 10% FBS (Gibco) and 1% penicillin/streptomycin (Gibco) during growth stages. Media supplied to the bioreactor was DMEM/F12 medium for all studies described. HEK293T cells were cultured in Opti-MEM Reduced Serum Medium (ThermoFisher) during transfection stages and during initial seeding in the bioreactor. NF*κ*B HEK293T cells were also used in studies and cultured in DMEM/F12 growth medium. NF*κ*B-GLUC-HEK293T cells were sorted to form a stable cell line after standard HEK293T cells were transfected with a NF*κ*B-GLUC-RFP lentivirus construct.

### 2.2 | Production of lentivirus particles

HEK cells were seeded in 10 cm dishes 24 h prior to plasmid addition to reach 80%–90% confluency at the time of plasmid addition. An initial study was performed (See Figure S1) that assessed the ability to seed cells and plasmids at the same time and the effect it had on transient transfection of lentivirus. For producing lentivirus progeny particles two solutions are used. Plasmid Solution A: 10 *μ*g lentivirus transfer vector pTK113-CMV-GFP, containing reporter green fluorescent protein (GFP) (Addgene) was combined with 10 *μ*g abm’s Second Generation (LV003) packaging mix (abm) in 1 mL Optimem. Plasmid Solution B: Dilute 80 *μ*L of LentiFectin^™^ Transfection reagent (G074 – abm) in 1 mL Optimem. Solutions A and B were incubated for at room temperature for 5 min before being mixed together well and incubated for 20 min at room temperature. The mixture was then added to cells in one of two ways: (1) to the 10 cm dish which had cells already seeded and incubated overnight at 37°C, or (2) with HEK cells seeded at that moment at 35%–40% confluency before adding the entire mixture to a 10 cm dish. The plasmid and HEK cell mix was seeded into the bioreactors and cells were allowed 4–5 h to adhere before beginning the flow of media.

### 2.3 | Seeding hollow fiber bioreactors

For all studies, Microkros 20-cm long, 0.2 *μ*m MWCO (molecular weight cut off), Polyethersulfone membrane (PES – hydrophilic) filter hollow fiber bioreactors (Repligen: C02-P20U-05-S) were used to seed HEK293T producer cells. The bioreactor fiber membranes had a surface area of 28 cm^2^, and 9 count of 0.5 mm fibers. All bioreactors came prepackaged and sterile. The bioreactor is composed of an outer lumen and inner lumen space separated by the membrane fibers. For loading the bioreactor with cell/plasmid suspension, 1 mL of volume was loaded in one unit with desired density of cells. The left most port on top of bioreactor was opened completely and right port was opened slightly to prevent pressure build up and allow flow through of cells/plasmid/media to the right side. Both ports were closed and side ports that access the inner lumen space were opened to connect to the inlet media pump and outlet stream to the fraction collector. Cell numbers of 2 × 10^6^ and 10 × 10^6^, giving densities of 0.071 × 10^6^ cells cm^−2^ and 0.36 × 10^6^ cells cm^−2^ on the 28 cm^2^ membrane surface, respectively, were tested as higher and lower end limits. The cell number 2 × 10^6^ was chosen as a starting point for testing as it has been used successfully in previous perfusions of mesenchymal stromal cells in these hollow fiber bioreactors,^[21–24]^ while 10 × 10^6^ was chosen in attempt to saturate the surface area with cells.

### 2.4 | Fraction collection set up

The general setup and operation of the perfusion system used in this paper are described in detail in our previous publications.^[25–27]^ A BioFrac fraction collector (Bio-Rad #7410002) was used to investigate the secretion dynamics over time from HEK293T cells in different conditions. All cell culture secretion time distribution experiments were performed using a multi-channel syringe pump (New Era Pump Systems NE-1600) to act as fresh media (DMEM/F12 – Gibco) reservoirs to be supplied to each bioreactor from 30 mL syringes with BD Luer-Lok Top (VWR). Tubing used to connect between the media syringe and the bioreactor, and from the bioreactor to the fraction collector was 1.6 mm Masterflex LS Platinum-Cured Silicone tubing (Cole Parmer – EW-96410–14), secured using female Luer connectors (Cole Parmer). Tubing was connected to the inner lumen space of the bioreactor using the side ports, so that anything collected in the fractions needed to permeate the fibers from the outer lumen space into the media-only inner lumen space. Multiple fraction collection channels could be set up in tandem by using a custom laser-cut multi-head dispenser.^[25]^ For all experiments, the multi-channel pump perfused each bioreactor with media at a flow rate of 0.25 mL h^−1^, amounting to 1 mL of media collected from a bioreactor in 4 h. Fractions were set to collect every 240 min (4 h) meaning that after every 4-h block of time, the fraction collector would move to the next position to collect the next sample. An average of 14 fractions were collected for each experiment, spanning a time frame of 56 h (2.3 days).

### 2.5 | TNF-*α*, sodium butyrate, and sodium dichloroacetate stimulation

For stimulation studies, we investigated the change in HEK293T production when in culture with inhibitory and stimulatory molecules. Specifically, for all stimulation experiments, the stimulant was mixed into the cell and plasmid suspension immediately before seeding into the bioreactor and allowed to slowly wash out during the perfusion. NF*κ*B HEK293T cells were first stimulated with 20 ng mL^−1^ TNF-*α* to activate the production of Gaussia Luciferase (GLuc). GLuc is a reporter protein that has minimal toxicity and is easily and quickly measurable in samples with high sensitivity.^[28]^ Sodium butyrate (NaBut) was added to HEK293T cells and plasmids before seeding in the bioreactor at 3 *μ*g mL^−1^, after testing ranges from 0 to 9 *μ*g mL^−1^ (Figure S4), and the stimulant was initially in the bioreactor but subsequently was washed out by the flow. Sodium dichloroacetate was added to HEK cultures before seeding at 10-, 20-, or 30-mM sodium dichloroacetate (DCA) in 2D (data not shown). Specifically, cells in culture with plasmids at the aforementioned ratios were plated in 6 cm dishes, and suspension amounts of 10, 20, or 30 mM DCA were added dropwise to each well and gently mixed. The cell, plasmid, and DCA mixture was subsequently added to the bioreactor for analysis. 30 mM DCA was used in bioreactor fraction collection studies.

### 2.6 | qPCR for titer

QPCR was used to determine the amount of virus (TU mL^−1^) for each experimental study group. The LV900 qPCR Lentivirus Titer Kit (abm) was used to determine quantity of lentivirus using a QuantStudio 3 Real-Time PCR system, 96-well, 0.2 mL (Applied Biosystems). Samples were assayed in triplicate.

### 2.7 | LDH assay

The lactate dehydrogenase assay (LDH) colorimetric kit (Abcam – ab102526) was used to measure LDH in collected media fractions as a marker of cell death. LDH is a cytosolic enzyme that is released into the cell culture medium upon damage to the plasma membrane. Briefly, fraction samples were vortexed after stabilizing to room temperature from freeze (−80°C) before adding 20 *μ*L to a 96-well plate in duplicate. Standard wells were set up in duplicate as indicated. Plate was measured using a kinetic loop in a plate reader (Varioskan LUX – Thermo Scientific), reading absorbance at OD 450 nm every 15 min for 1 h total.

### 2.8 | Gaussia luciferase assay

Samples of each fraction were added at 20 *μ*L in duplicate to a 96-well black walled flat bottom plate (Corning). The gLuc substrate coelenterazine (Nanolight) was diluted in PBS (1 *μ*L per mL PBS). Substrate was added to plate in intervals so that plate and substrate could be left in the dark when not in use. Plate reader (Varioskan LUX – Thermo Scientific) was set up to run the plate backwards and forwards with releasing the plate at every interval so that substrate could be added, and plate put back into the plate reader.

### 2.9 | Transduction studies

HEK293T target cells were seeded in wells of a 96-well plate at 10,000 cells in 200 *μ*L 24 h prior to addition of fraction samples. After other analyses were performed on each of the fraction samples, fractions were spun down in high-speed benchtop centrifuges (Centrifuge 5424 – Eppendorf) at 20,000 × *g* for 90 min at which point a visible lentivirus pellet had formed on the side of the Eppendorf tubes. Media was aspirated and pellet was resuspended in 200 *μ*L of serum free Optimem for transduction. A total of 10 *μ*g mL^−1^ polybrene was added to each well to enhance transduction. The 200 *μ*L of media was added to one well of the 96-well plate and after 24, 48, or 72 h GFP expression was measured using a plate reader (Varioskan) and reading fluorescence at 488 excitation and 518 emission.

### 2.10 | Fluorescent microscopy

An Axio Observer Light Microscope (ZEISS) was used to qualitatively analyze samples for expression of the green fluorescent protein (GFP).

### 2.11 | Flow and image cytometry

Lentiviral transduction of target HEK cells was analyzed by GFP expression using flow cytometry analysis performed using a BD FACS Canto II and FACS Diva software (BD Biosciences). Data was analyzed using FlowJo Software (Tree Star). 30,000 and 50,000 events per sample were collected depending on experiment. Positive staining was gated based on negative control cells that did not express GFP. Images for groups were acquired after 72 h using an image cytometer (Nexcelom Celigo) using the brightfield channel in combination with the green channel (483/536) to detect GFP+ cells. The Celigo Expression Analysis application was used with target 1 (brightfield) + 2 (GFP).

### 2.12 | Statistics

Results are represented as mean ± SD. All experiments were run in triplicate unless otherwise indicated and statistically significant comparisons between each group were made using one- or two-way ANOVA followed by Tukey’s post hoc test or test for multiple comparisons. Mixed effects analysis was used in cases where datasets had missing replicates. *p* < 0.05 was considered significant. GraphPad Prism was used for graphing and measuring significance.

## 3 | RESULTS AND DISCUSSION

### 3.1 | Investigating secretion dynamics of HEK293T producer cells at different densities

**FIGURE 2:**
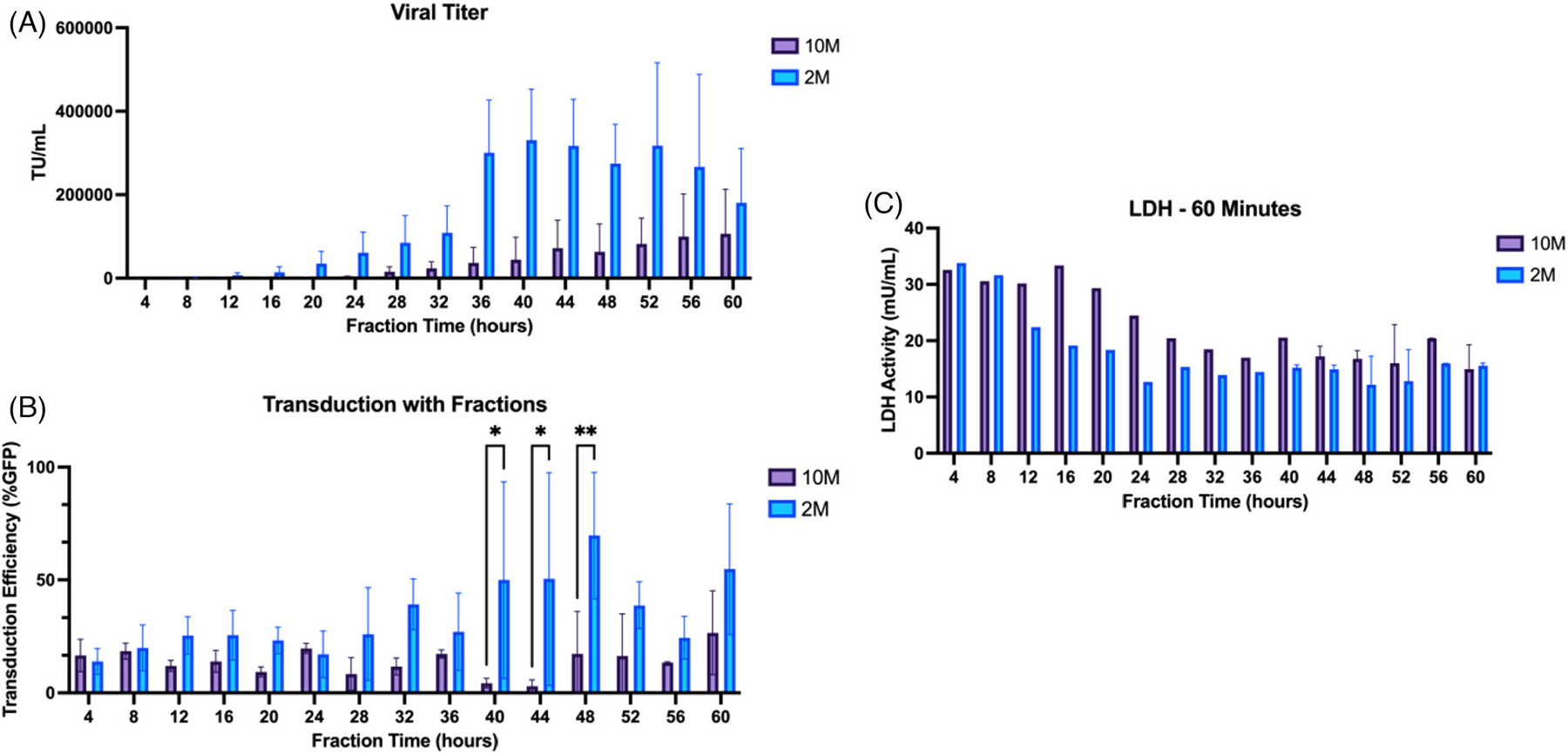
Comparison of titer, transduction efficiency of fractions and LDH of 2 × 10^6^ and 10 × 10^6^ density HEK293T producer cells in bioreactors over 60 h. Secretion dynamics of producer HEK293T cells in a hollow fiber bioreactor at two different densities was measured. (A) Total quantities of lentiviral particles produced over 60-h was quantified using qPCR. Peak production onset began around 32–36 h. The two groups were not statistically significantly different from each other at any one time point. (B) Transduction efficiency of fractions after 72 h in culture as measured using green fluorescence at Ex. 488 and Em. 518. Fractions at each time point were centrifuged at 20,000 × *g* for 90 min, and virus pellet was resuspended in Optimem before combining with 10,000 target HEK cells and 10 ***μ***g/mL polybrene in a 96-well plate. *N* = 3 per each seeding density group. Results are represented as mean ± SD. (*p < 0.05, **p < 0.01.) (C) LDH activity was measured for each time point fraction sample for the 2 × 10^6^ (2 M) and 10 × 10^6^ (10 M) seeding density groups. LDH was quantified in a plate reader at OD 450 nm. Plates were set up in duplicate; timepoints up to 36 h have only one successful replicate. Results are represented as mean ± SD.

Initial studies were performed with two different HEK293T producer cell densities (2 × 10^6^ and 10 × 10^6^ cells on hollow fiber membranes with 28 cm^2^ of surface area) to compare the difference in secretion dynamics over a 2-day period. 2 × 10^6^ and 10 × 10^6^ HEK cells and plasmid mix were seeded in bioreactors in triplicate. Contrary to traditional 2D transient transfection, HEK and plasmids were seeded simultaneously after previous studies showed that this did not negatively impact transfection (Figure S1). Fractions of HEK cell secretions at 2 × 10^6^ and 10 × 10^6^ cells were collected over a 60-h span at 4-h time intervals. As shown in Figure 2A, fractions collected at later time points had higher quantities of detectable lentivirus as quantified by qPCR with peak production onset occurring around 32–36 h. The tubing downstream of the bioreactor was left empty prior to starting the perfusion so that the first media collected in the fractions was the media initially in the bioreactor. At each time point, the 2 × 10^6^ seeding density group appeared to have higher viral titer values than the 10 × 10^6^ density group, although the difference between the two density groups at any given timepoint was not statistically significant. For the 10 × 10^6^ group, titer rose steadily beginning at 32–36 h until the end of the 60h period, but titer never met the titer of the 2 × 10^6^ groups. Fractions were tested for their ability to transduce secondary target HEK cells at equivalent MOIs (virus to cell ratio), as a measure of the potency of the virus collected at each fraction, and it appeared as though fractions with higher viral titer had higher transduction efficiency of target cells, seen at the later time points after the 36-h mark, although differences in titer were not statistically significant (Figure 2B). MOI was maintained using specific volumes of each viral fraction that corresponded to an equal number of particles to each well (for example, if one fraction was 1 × 10^5^ we would use 100 *μ*L, and if another fraction was 5 × 10^5^ we would use 20 *μ*L (1 × 10^4^ particles each) to maintain equal MOI. All fractions from the 2 × 10^6^ group were able to transduce cells to some degree above 20% GFP expression. In the 10 × 10^6^ group, transduction efficiency of fractions was constant for all fraction times around 20% or lower, with a peak efficiency seen at the 60-h around 35%. Representative images of transduced cells in each group are shown in Figure S2. We hypothesized that there was a reduction in the lentiviral particle output at the higher cell density because the cells may form a barrier that limits the transfer of particles into the hollow fibers where the fractions were collected from. Additionally, we looked at cell lysis over time via the LDH assay, which showed from Figure 2C that early fractions had much higher LDH activity (mU mL^−1^) than at later time points in both cell groups. Although these data seem to be inversely proportional to the titer data, further experimentation will be needed to determine whether LDH was high in the beginning due to cell death from stress of the seeding process. All subsequent experiments utilized the 2 × 10^6^ seeding density for producer HEK293T cells to assess how different parameters impact the LV production time course of transiently transfected HEK cells.

### 3.2 | Secretion dynamics with an NF*κ*B promoter inflammatory reporter cell line

**FIGURE 3:**
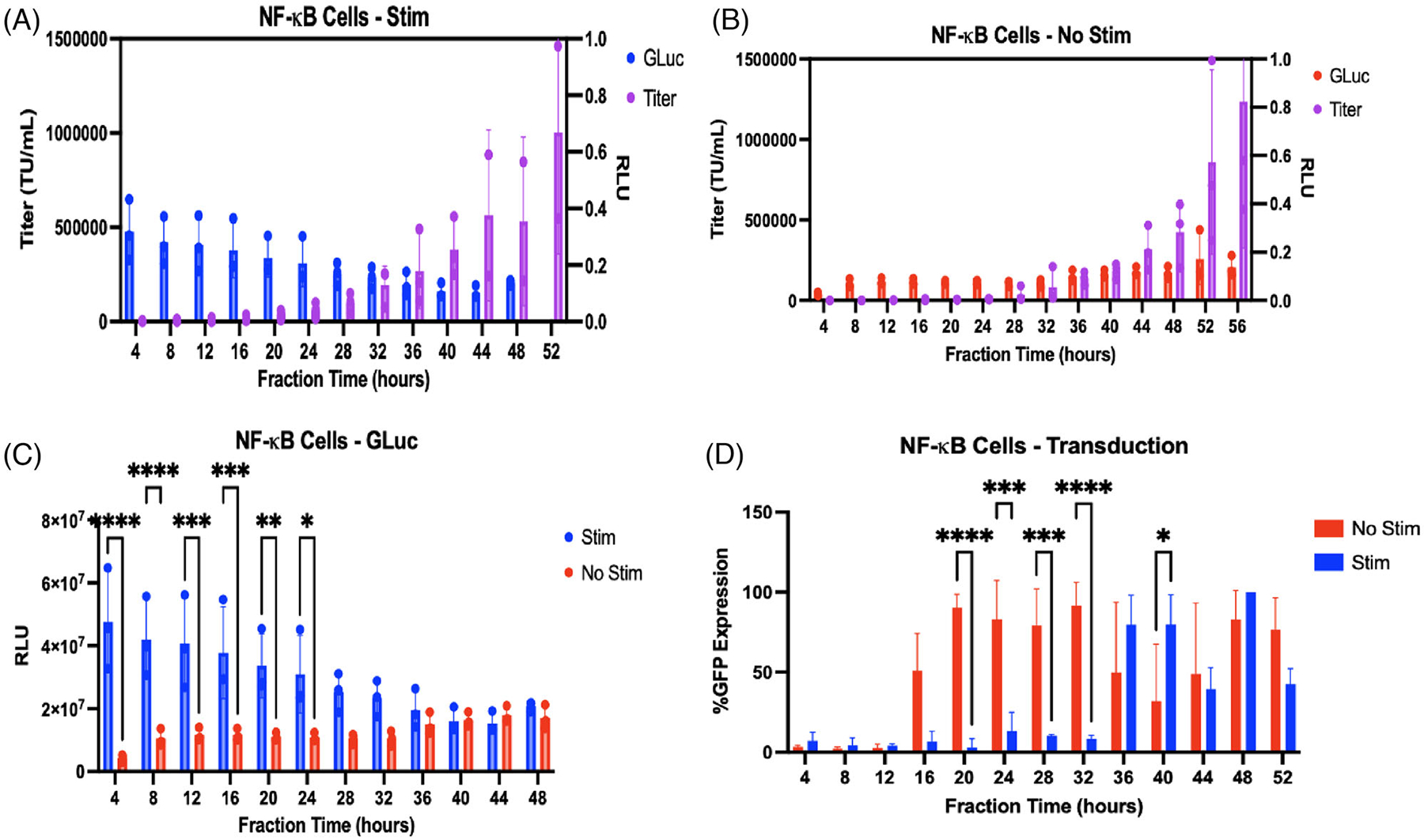
NF*κ*B-driven GLuc secretion rates report inflammatory state from both TNF-*α* stimulation and the presence of viral components. NF*κ*B-GLuc HEK293T cells were seeded with plasmid complex and stimulated with 20 ng mL^−1^ TNF-*α* in bioreactors at *N* = 3. *N* = 3 bioreactors were also seeded with non-stimulated NF*κ*B-GLuc HEK293T cells. (A) Onset of viral production and peak production (32–36 h) in stimulated NF*κ*B cells occurred with reduced immune response as measured by GLuc secretion from NF*κ*B activation with TNF-*α*, (B) Upon onset of viral production, non-stimulated NF*κ*B cells showed a slight increase in GLuc activity, likely triggered by the LV particles themselves. *N* = 3 was used for each seeding density group. Results are represented as mean ± SD. (C) Significantly higher GLuc secretion was seen in stimulated groups over non-stimulated controls, and GLuc secretion provided a good read out on the inflammatory status of the cells in culture. *N* = 3 was used for each seeding density group. Results are represented as mean ± SD. Two-way ANOVA with multiple comparisons were used to determine significance (*p* < 0.05). (D) Transduction efficiency of fractions in NF*κ*B HEK stimulated and non-stimulated groups as measured by GFP expression. In alignment with titer results, later fractions from the stimulated group had higher transduction efficiency of target cells. In the non-stimulated group, transduction efficiency of target cells was significantly higher in earlier time point fractions between 20 and 32 h. Results are represented as mean ± SD and multiple unpaired t-tests were used to determine significance (*p* < 0.05).

Next, we investigated the relationship between the intracellular immune response and the LV production timecourse. We utilized HEK293T cells engineered with a secreted luciferase reporter (GLuc) driven by a promoter with the NF*κ*B response element (NF*κ*B -GLuc). NF*κ*B regulates innate immunity and responds to viral antigens by binding to NF*κ*B response elements in the promoters pro-inflammatory genes. Thus, NF*κ*B drives the expression of GLuc, which is secreted into the flow at a rate dependent on the inflammatory state of the cells. We wanted to test whether an intracellular immune response in HEK cells is activated by transient transfection similarly to activation of NF*κ*B through TNF-*α* stimulation.^[29,30]^ We also included groups stimulated with TNF-*α* at 3 *μ*g mL^−1^ prior to being seeded (Figure 3A,B) to allow us to test whether TNF-*α*-induced inflammation impacts LV production dynamics. Titer was plotted alongside GLuc readout for each group to assess the effects of immune activation on LV production. Figure 3A shows the onset of LV production occurred around the 32–36-h mark as seen with the previous experiments (Figure 2), coinciding with the drop in GLuc secretion. The titer of the stimulated group rose steadily after the 32–36-h timepoint. Notably, the titer from the 2 × 10^6^ NF*κ*B-Gluc HEK293T cells was higher than the results seen previously with unmodified HEK293T cells at all timepoints. This could be due to the fact that NF*κ*B-Gluc cell line had previously been engineered with viral machinery, making subsequent production of LVs easier through activation of some tolerance mechanism. From Figure 3B, the onset of production in the non-stimulated group also occurred at the 32–36-h timepoint and interestingly, following this onset, there was a slight increase seen in GLuc secretion, even though these cells were not directly stimulated with a pro-inflammatory agent. Thus, production of LVs in HEK293T cells appears to weakly trigger NF*κ*B activation but does not impact LV yields. Figure 3C compares the stimulated and non-stimulated groups.

We next measured the potency of LVs in each fraction produced by the stimulated and non-stimulated NF*κ*B cell groups. Figure 3D shows there was a significant increase in GFP expression in target cells transduced with lentivirus from the non-stimulated group at timepoints between 20 and 32 h, with the majority of target cells transduced with these fractions having nearly 100% expression. The delayed onset of transduction of target cells for the stimulated group could be due to the inflammation stimulus causing production of poor quality virus (defined here as fraction samples that contain a high percentage of damaged or empty virus that is not infective and thus has low transduction efficiency into target cells). This suggests that the addition of an anti-inflammatory to the culture could help produce potent virus.

### 3.3 | Cell cycle arrest with sodium butyrate (NaBut)

**FIGURE 4:**
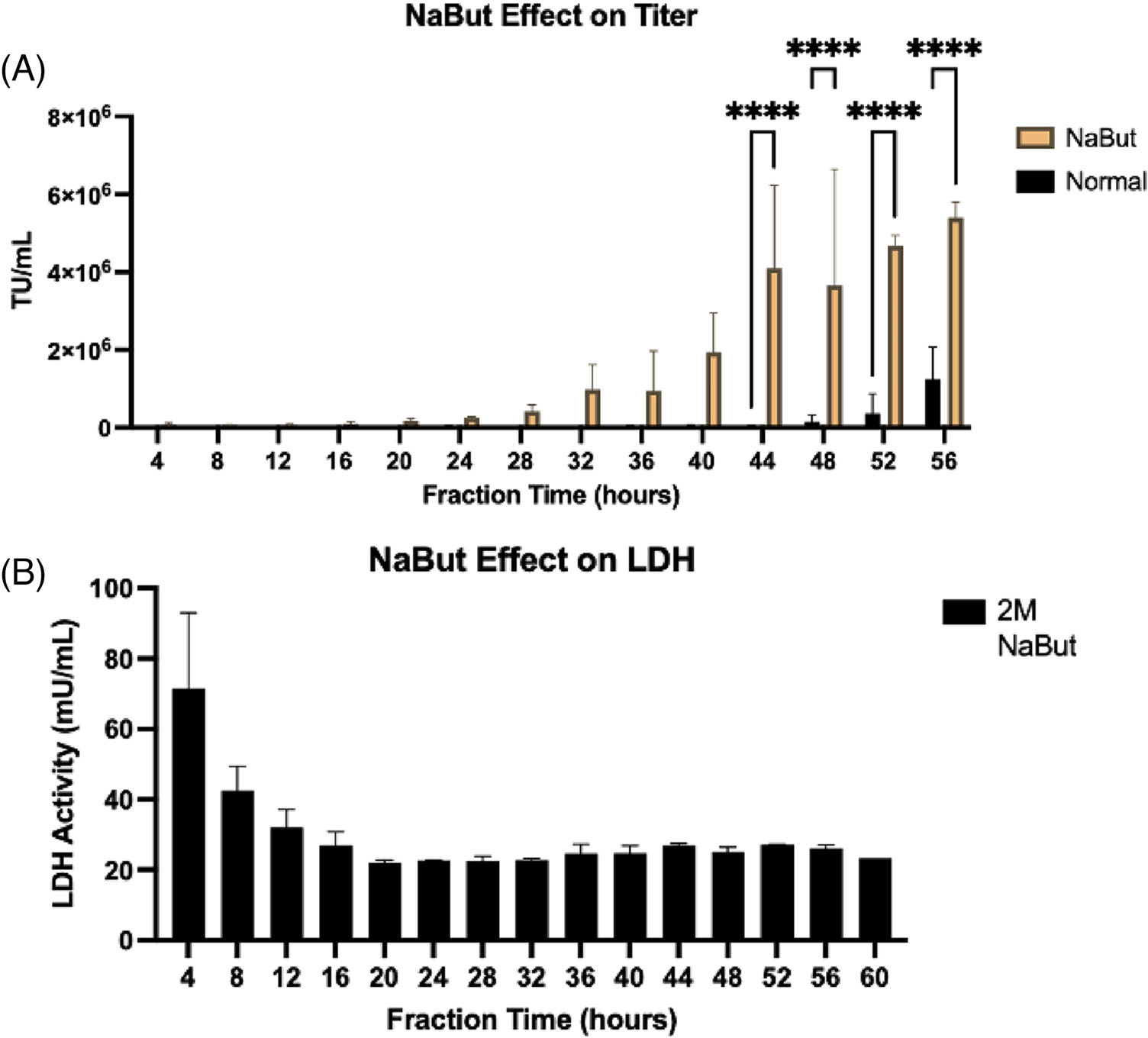
Effect of sodium butyrate stimulation on titer and LDH from HEK293T cells. HEK293T cells were seeded in hollow fiber bioreactors in triplicate either with or without the cytostatic agent sodium butyrate. (A) Titer was determined at each fraction collection time point. (B) LDH activity from cell supernatant in each fraction was quantified to determine the impact of stimulation on the HEK cell death. Results are represented as mean ± SD. Two-way ANOVA was used with multiple comparisons to determine significance (*p* < 0.05).

We next tested the effect of a cell cycle arresting agent on LV production dynamics. Sodium butyrate (NaBut) is a histone deacetylase inhibitor that has been shown to enhance transient gene expression in HEK cells by suppressing cell proliferation.^[31]^ NaBut arrests cells during the G1 phase of the cell cycle where cells are larger and more metabolically active. First, we tested a several concentrations of NaBut in 2D culture using a range of literature values (1, 3, 6, and 9 *μ*g mL^−1^) and measured GFP expression in target cells after 72 h.^[32–34]^ We found the highest GFP expression (80%) with 3 *μ*g mL^−1^ NaBut (Figure S4) and used this concentration in subsequent bioreactor studies. Triplicate experimental and control bioreactors were seeded with 2 × 10^6^ transfected HEK293T cells either with or without NaBut. Figure 4A shows there was a significant increase (*p* < 0.01) in the titer in the NaBut group after 40 h compared to normal HEK cells, and a higher titer at all timepoints compared to normal cells, with a >10-fold increase at the highest points. Interestingly, production onset was earlier in the NaBut group. This NaBut-related LV production increase is consistent with literature.^[32–34]^ Next, we tested the effect of NaBut on LDH release from cells and transduction efficiency of fractions. Figure 4B shows there was high LDH activity in the first 8 h in the NaBut-containing bioreactors, but after 20 h the activity decreased to around 20 mU mL^−1^ and remained constant. LDH activity did not reveal any negative impact of NaBut on cell viability, which is corroborated by our 2D studies where we saw that there was no negative impact on cell viability with cells cultured with NaBut for 72 h.

### 3.4 | Downregulation of the PDK pathway with sodium dichloroacetate (DCA)

**FIGURE 5:**
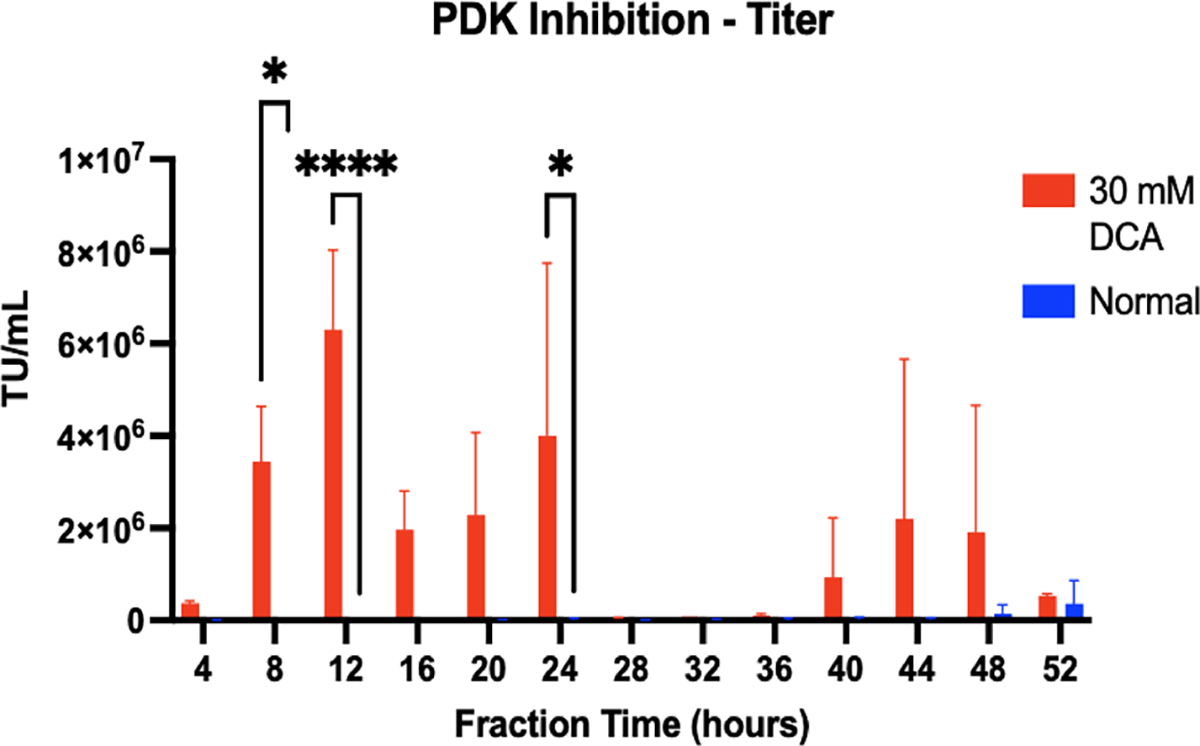
Early onset of viral secretion from HEK cells stimulated with DCA to downregulate PDK. 30 mM DCA was added to transiently transfected HEK cell cultures in bioreactors (*N* = 3) and titer was compared to normal HEK cells. Virus production peaked during early time points between 8 and 24 h when HEK cells were stimulated with DCA. There was a period of low production between 28 and 36 h, and production was measurable again in fractions after 40 h. Compared to control HEK cells, virus production was significantly higher at early time points, and was higher but not significant at time points after 24 h. Results are represented as mean ± SD. Two-way ANOVA was used with multiple comparisons to determine significance (*p* < 0.05).

Finally, we tested the effects of an agent that downregulates the pyruvate dehydrogenase kinase (PDK) which studies have shown increases titer and reduces lactate accumulation in recombinant retrovirus production processes.^[35,36]^ We tested a range of concentrations of the PDK inhibitor sodium dichloroacetate (DCA) from the given citations to determine the optimal range for enhanced LV production in HEK293T cells in the bioreactor.^[35]^ Figure 5 shows that 30 mM DCA stimulation in HEK cells resulted in earlier onset of LV secretion between 8- and 24-h timepoints. The mechanism described in the literature^[35]^ suggests that the DCA leads to an early reduction in lactate accumulation by redirecting pyruvate into the TCA cycle for energy production, as well as recruitment of other metabolic pathways, and that this leads to the earlier onset of secretion seen in the titer compared to nFon-stimulated controls. There was also a low production period between 24 and 40 h, which could be a result of DCA washout and the period of time where cells returned to normal metabolic functioning to begin producing virus again after 40 h at comparable rates to normal HEK cells.

### 3.5 | Effect of prior viral transduction of cell line on titer

In addition to our perfusion experiments, we investigated how prior viral vector transduction of a cell line impacts its LV production. We compared LV production rates of an engineered HEK293 line (EF1*α*-GLuc-IRES-RFP) to HEK293T cells and found that the previously engineered cells had higher titer, but the difference was not statistically significant (Figure S3). This may indicate that cell lines previously exposed to lentiviral components during genetic modification may develop a tolerance to them, allowing them to produce the LVs more easily. This could be relevant to understanding the productivity increases seen in stable packaging lines engineered using LVs.^[37,38]^

## 4 | CONCLUDING REMARKS

Here, we provided a novel view into the dynamics of LV production dynamicsonanhourlytimescaleusinghollowfiberbioreactorsinasimple, one-way perfusion system. We revealed how known interventions impact production dynamics and discussed how this knowledge could help engineers to optimize their application of these interventions to maximize LV yield.

## Supplementary Material

Supplementary Figures

## Data Availability

The data that support the findings of this study are available from the corresponding author upon reasonable request.

## Abbreviations:

DCA: sodium dichloroacetate
GFP: green fluorescence protein
gLuc: Gaussia luciferase
HEK: human embryonic kidney
LDH: lactate dehydrogenase
LV: lentivirus
MOI: multiplicity of infection
NaBut: sodium butyrate
NF: nuclear factor
PDK: pyruvate dehydrogenase kinase
TNF: tumor necrosis factor
TU: transduction units
