# Supplementary material for: Investigating dynamics of lentiviral vector secretion from HEK293T producer cells using a fractionated perfusion system
Source: Biotechnol J. Author manuscript; Available in PMC 2024 Jul 31. (PMC11289840; doi:10.1002/biot.202300097)
Supplement: Supplementary Figures [file NIHMS2007845-supplement-Supplementary_Figures.pdf]

Supplementary Figures

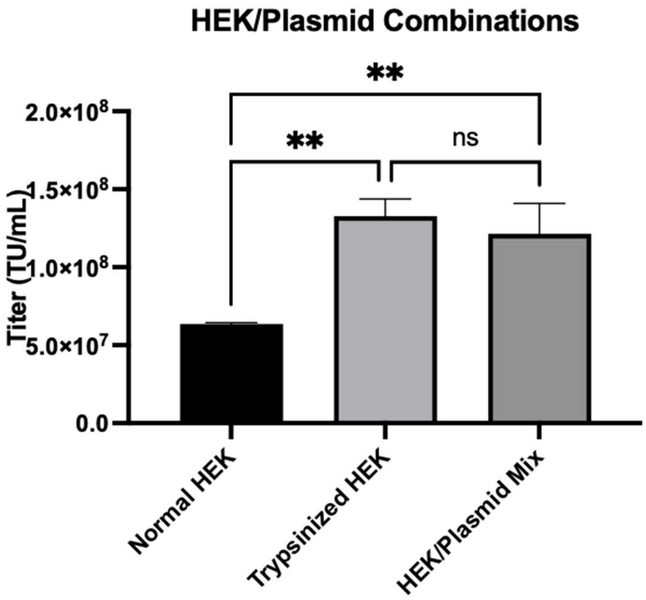

Supplementary Figure 1 Seeding HEK and plasmids for transient transfection under different conditions. In order to streamline the seeding of transiently transfected HEK cells into hollow fiber bioreactors, HEK293T producer cells were transfected with a lentivirus plasmid complex under various conditions. Normal HEK refers to the standard procedure of seeding HEK cells 24 hours prior to plasmid addition in a 10 cm dish to reach 80-90% confluency on day of plasmid addition. The trypsinized HEK group was set up the same as the normal group, however the cells once plasmid was added, were trypsinized, centrifuged and pelleted, and resuspended in fresh media to be plated in a new 10 cm dish to adhere again and start producing virus. Lastly, the HEK/plasmid mix was the direct seeding of HEK cells and plasmid mix together in the 10 cm dish on the same day and allowing to adhere. N=3 replicates per group. qPCR was used to determine titer for each group (TU/mL).

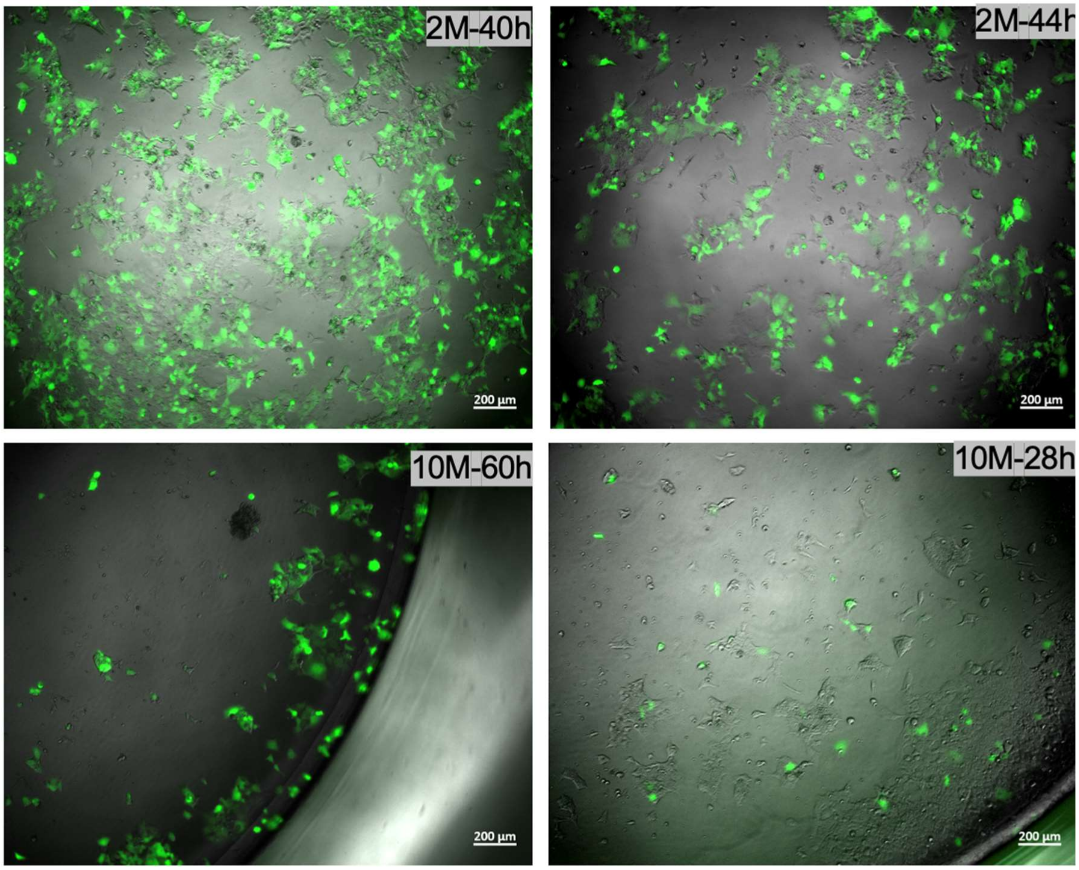

Supplementary Figure 2. Transduced HEK cells from 2x10^6 and 10x10^6 fraction groups. 2x10^6 (2M) and 10x10^6 (10M) HEK293T producer cells were seeded in hollow fiber bioreactors for 60 hours and fractions were collected every 4 hours. Each fraction contained lentiviral particles, that were concentrated and put with target cells to measure transduction efficiency after 72 hours. Representative images from 2M (A-B) and 10M (C-D) groups. 2M groups had nearly 100% transduction in the 40–44-hour fractions shown, while there was much less GFP expression seen in 10M fractions at hour 28 and 60. Images taken using ZEISS microscope. Scale bar: 200 μm. N=3 per seeding density group.

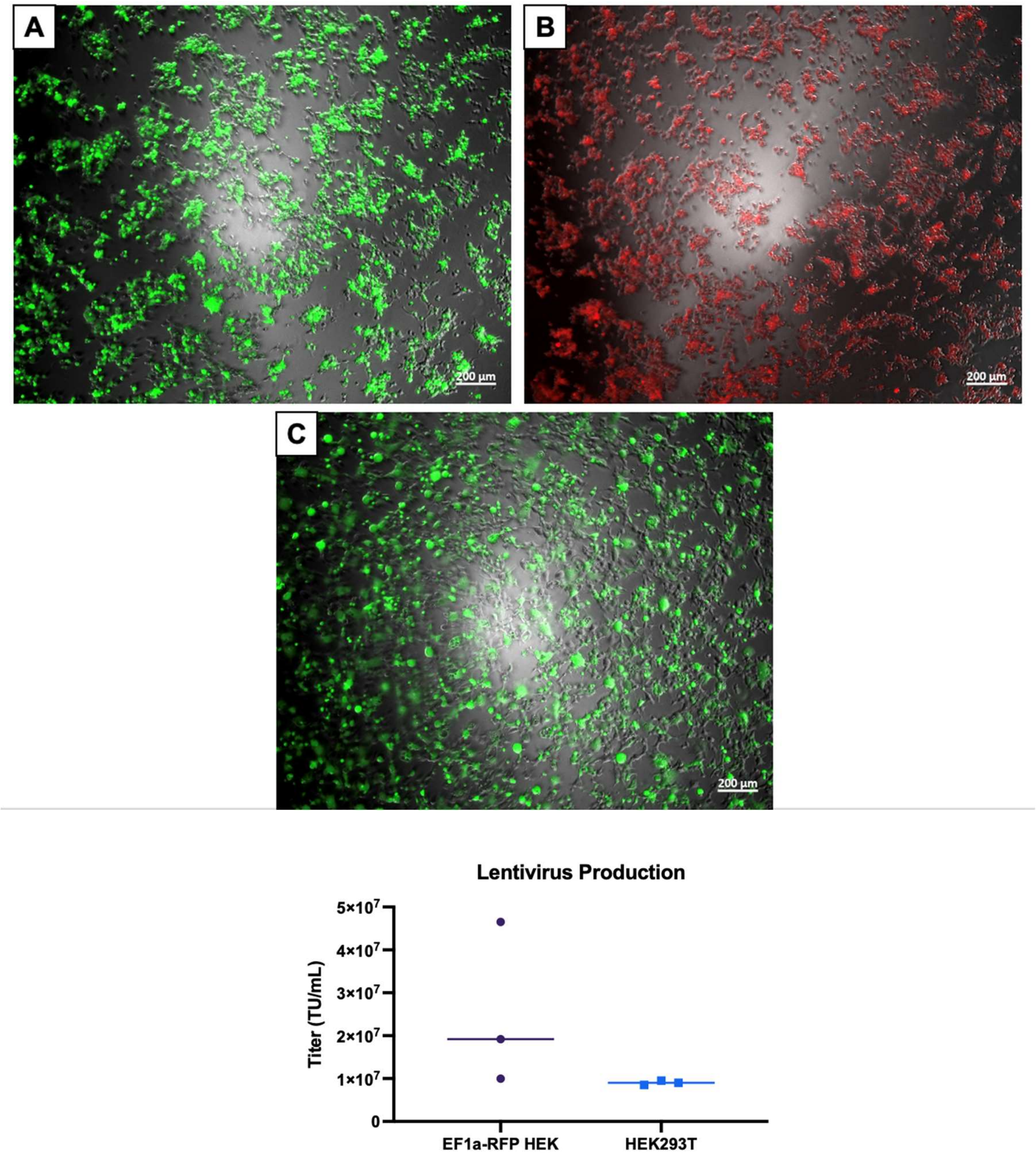

Supplementary Figure 3. Transiently transfected engineered HEK cells. (A) HEK cells previously engineered to express RFP with an EF1α promoter was transiently transfected in 6-wells in triplicate. 48 hours after, images were taken using ZEISS microscope. Cells expressing GFP. (B) The same cells expressing RFP. Both expressions are equally bright, and all cells are transiently expressing both plasmids. (C) HEK293T cells transfected with the same GFP construct. Scale bar: 200 μm. (D) Engineered HEK cells and normal HEK cells were seeded in 6-well plates in triplicate. Production of lentiviral particles was measured using qPCR. Results are represented as mean $\pm$ SD. Unpaired t-test was used to determine significance ($p < 0.05$).

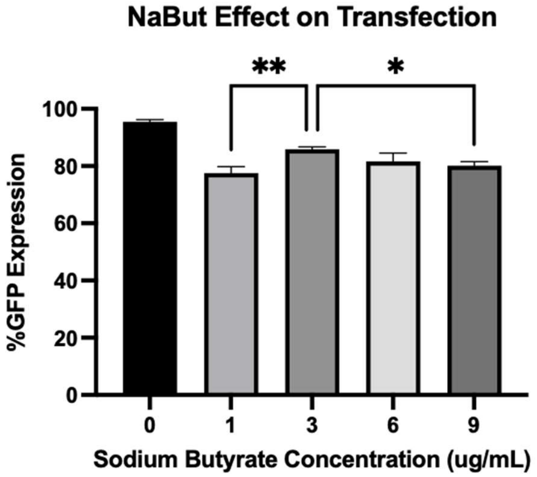

Supplementary Figure 4. Testing various concentrations of Sodium Butyrate and the effect on transduction. HEK cells were transduced in 6-wells in triplicate with GFP virus, and 48 hours after, production of lentiviral particles was measured using qPCR. Results are represented as mean ± SD. One-way ANOVA was used to determine significance (*$p < 0.05$, **$p<0.01$).
